# High maternal mortality rate associated with advanced maternal age in Japan

**DOI:** 10.1038/s41598-023-40150-4

**Published:** 2023-08-09

**Authors:** Hiroaki Tanaka, Junichi Hasegawa, Shinji Katsuragi, Kayo Tanaka, Tatsuya Arakaki, Masamitsu Nakamura, Eijiro Hayata, Masahiko Nakata, Takeshi Murakoshi, Akihiko Sekizawa, Isamu Ishiwata, Tomoaki Ikeda

**Affiliations:** 1 Department of Obstetrics and Gynecology, Mie University School of Medicine, 2-174 Edobashi, Tsu, Mie 514-8507 Japan; 2 Department of Obstetrics and Gynecology, St. Marianna University School of Medicine, Kanagawa, Japan; 3 Department of Obstetrics and Gynecology, Miyazaki University School of Medicine, Miyazaki, Japan; 4 Department of Obstetrics and Gynecology, Showa University School of Medicine, Tokyo, Japan; 5 Department of Obstetrics and Gynecology, St. Marianna University Yokohama Seibu Hospital, Kanagawa, Japan; 6 Department of Obstetrics and Gynecology, Toho University Omori Medical Center, Tokyo, Japan; 7 Department of Obstetrics and Gynecology, Seirei Hamamatsu General Hospital, Shizuoka, Japan; 8 Department of Obstetrics and Gynecology, Ishiwata Obstetrics and Gynecology Hospital, Ibaraki, Japan

**Keywords:** Diseases, Health care

## Abstract

This study aimed to clarify the relationship between maternal mortality and advanced maternal age in Japan and to provide useful information for future perinatal management. Maternal death rates by age group were investigated for all maternal deaths in Japan for an 11-year period, from 2010 to 2021. Maternal deaths among those aged ≥ 40 years were examined in detail to determine the cause, and the number of deaths by cause was calculated. The causes of onset of the most common causes of death were also investigated. The maternal mortality rates were 0.8 (95% confidence interval [CI] 0.3–4.7) for < 20 years, 2.6 (95% CI 1.7–3.8) for 20–24 years, 2.9 (95% CI 2.3–3.6) for 25–29 years, 3.9 (95% CI 3.3–4.5) for 30–34 years, 6.8 (95% CI 5.9–7.9) for 35–39 years, and 11.2 (95% CI 8.8–14.3) for ≥ 40 years of age. Patients who were ≥ 40 years of age had a significantly higher mortality rate compared to that in other age groups. Hemorrhagic stroke was the most common cause of death in patients aged ≥ 40 years (15/65 [23%]), and preeclampsia (8/15 [54%]) was the most common cause of hemorrhagic stroke. Maternal mortality is significantly higher in older than in younger pregnant women in Japan, with hemorrhagic stroke being the most common cause of maternal death among women > 40 years of age. More than half of hemorrhagic strokes are associated with hypertension disorder of pregnancy. These facts should be considered by women who become pregnant at an advanced age and by healthcare providers involved in their perinatal care.

## Introduction

There has been an increase in the number of older women who are pregnant, and advanced maternal age (AMA) has been increasing yearly worldwide^1–5^. With increasing age of pregnancy, there is an increase in various maternal complications^5–7^. A study done in Japan reported that AMA increases the need for cesarean section and the risk for preeclampsia, placenta previa, and preterm delivery^6^. It has also been reported that AMA increases the risk of cervical incompetence, gestational diabetes, preterm water breaking, and preterm delivery^5,7^.

AMA has been reported to increase various maternal complications as well as maternal mortality^3^. It is predicted that the number of pregnancies at older ages will increase in the future owing to advances in assisted reproductive technologies and lifestyle changes. However, the relationship between age and maternal mortality in Japan has never been studied. This study aimed to clarify the relationship between maternal mortality and AMA in Japan and to provide useful information that will contribute to future perinatal management.

## Materials and methods

### Maternal mortality data

All maternal deaths in Japan during the 11-year period from 2010 to 2021 were included. Information on maternal deaths was obtained from data registered in the Japan Maternal Death Exploratory Committee (JMDEC) of the Japan Association of Obstetricians and Gynecologists (JAOG), which was launched in 2010 and covers all maternal deaths in Japan^8^.

The JMDEC consists of 15 obstetricians, four anesthesiologists, two emergency physicians, two pathologists, and medical specialists and forensic experts. The registration system has been described in previous reports^8–17^.

When a maternal death occurs, the attending physician registers the case with the Japanese Society of Obstetrics and Gynecology and sends a report to the JAOG. The report consists of 22 pages and approximately 100 questions and is intended to elicit detailed information about the clinical course, facility characteristics, and staff who participated in the patient's care, along with medical records such as anesthesia records, medical images, laboratory data, pathology reports, and autopsy reports.

All personal information contained in reports and medical records, such as maternal characteristics, clinicians, and facility geography, are anonymized in the JAOG. All of these anonymized data from across Japan are sent to the JMDEC, where they are analyzed and discussed at the JMDEC's monthly meetings regarding factors associated with maternal mortality and the circumstances of death.

### Age groups

Registered maternal deaths were categorized by age: < 20, 20–24, 25–29, 30–34, 35–39, and ≥ 40 years, and the maternal mortality rate was calculated for each age group.

Maternal mortality rates were also calculated for each age group by the major cause of death in Japan. Maternal mortality rate is the number of maternal deaths per 100,000 births. No statistical adjustment was made because the population was almost exclusively Japanese, there were no racial differences, and there was no disparity in access to medical care due to the national medical insurance and free maternal health checkup systems.

### Maternal deaths among pregnant women aged 40 years and older

Maternal deaths among women aged ≥ 40 years were examined in detail to determine the causes of death, and the number of deaths by cause was calculated. The causes of onset were also investigated for diseases leading to the highest number of deaths.

### Statistical analysis

Statistical analyses were performed to determine perinatal mortality using JMP 13.2.0 (SAS Institute Inc., Cary, NC, USA). Unless otherwise specified, univariate statistics and 95% confidence intervals (CI) were evaluated for the primary endpoint, perinatal mortality. Comparisons of perinatal mortality were performed using the Mann–Whitney U test. Statistical significance was set at *p* < 0.05. The Mann–Whitney U test was used for two-group comparisons and adjustments for multiple comparisons were made using the Bonferroni test. P-values for comparisons of maternal mortality rates between age groups were calculated using the Kruskal–Wallis test. *P*-values for maternal mortality rate comparisons between the 40 years and older and other age groups for each cause of death were calculated using the Mann–Whitney U test and adjusted for using the Bonferroni test.

### Ethics approval and consent to participate

This study was approved by the ethics boards of the National Cerebral and Cardiovascular Center, Osaka, Japan, and JAOG. This study was conducted in accordance with the principles of the Declaration of Helsinki. Informed consent was not obtained from patients or their families because this study was based on the analysis of institutional forms, and patient records and information were anonymized prior to the analysis. Additionally, under the conditions of approval for "medical and biological research involving human subjects" in Japan informed consent was not required for this study^18^, and JAOG approved that informed consent was not required for this study.

## Results

### Overall maternal deaths and international classification of diseases maternal mortality (ICD-MM) classification system

Between 2010 and 2021, 512 maternal deaths were registered in the Maternal Death Registration Project. The number of births in Japan during the same period was 11,557,975 cases. Of the 512 maternal deaths, 335 (65.4%) were direct deaths and 150 (29.3%) were indirect deaths. Twenty-seven (5.3%) were unclassifiable.

Overall, 497/512 (97.1%) cases could be classified according to the World Health Organization (WHO) ICD-MM. The breakdown of the classification is as follows: Group 1, 4 cases (0.8%); Group 2, 47 cases (9.5%); Group 3, 88 cases (17.7%); Group 4, 28 cases (5.6%); Group 5, 126 cases (27.3%); Group 6, 8 cases (1.6%); Group 7, 133 cases (26.8%); Group 8, 45 cases (9.1%); and Group 9, 8 cases (1.6%).

### Number of deaths and maternal mortality rates by age group

**Figure 1:**
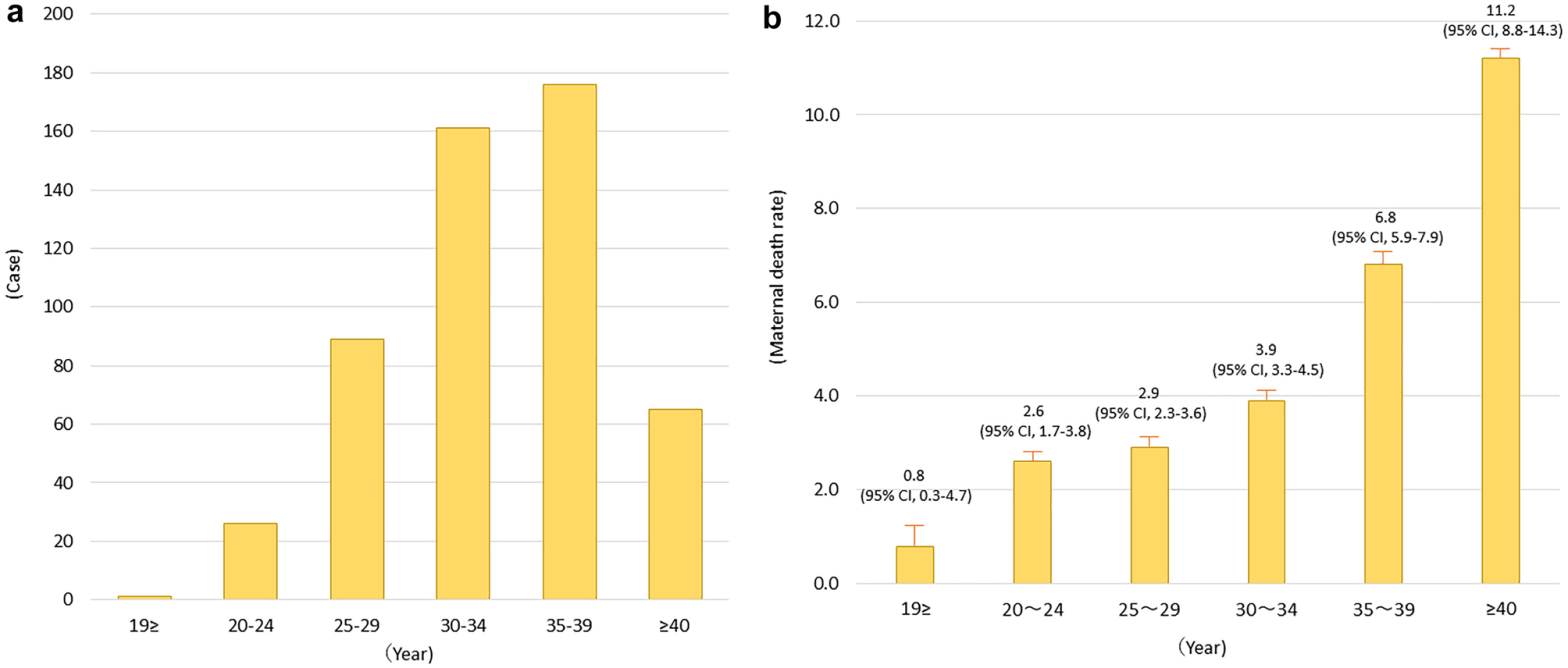
(**A**) Maternal deaths by age group. (**B**) Maternal mortality rates by age group.

The number of maternal deaths by age group was as follows: < 20 years, 1 case; 20–24 years, 26 cases; 25–29 years, 89 cases; 30–34 years, 161 cases; 35–39 years, 176 cases; and 40 years, 65 cases (Fig. 1A).

Maternal mortality rates by age group are shown in Fig. 1B. The maternal mortality rates were 0.8 (95% CI 0.3–4.7) for those < 20 years, 2.6 (95% CI 1.7–3.8) for those 20–24 years, 2.9 (95% CI 2.3–3.6) for those 25–29 years, 3.9 (95% CI 3.3–4.5) for those 30–34 years, 6.8 (95% CI 5.9–7.9) for those 35–39 years, and 11.2 (95% CI 8.8–14.3) for those ≥ 40 years of age. Pregnant women ≥ 40 years of age had significantly higher mortality rates compared to that in other age groups (*P* < 0.01). The maternal mortality rates by cause are shown in Table 1.

**Table 1:** Maternal death rate for each causative disease according to age group.

Age (years)	19** ≥ **	20–24	25–29	30–34	35–39	** ≥ **40
Obstetric hemorrhage	–	0.15	0.49	1.18	2.14	1.44
Amniotic fluid embolism	–	0.15	0.31	0.54	1.26	1.26
Hemorrhagic stroke	–	0.59	0.72	0.61	1.15	2.69
Pulmonary embolism	–	–	0.54	0.44	0.66	1.08
Infection	–	0.44	0.36	0.57	0.60	0.90
Cardiovascular disease	0.86	0.59	0.36	0.54	0.60	0.72
Suicide	–	0.44	0.31	0.34	0.93	1.44
Malignant disease	–	–	0.09	0.37	0.49	0.36
Other	–	0.88	0.40	0.47	0.71	0.90
Unknown	–	0.59	0.40	0.41	0.99	0.90

### Maternal mortality in the 40 years and older group

**Figure 2:**
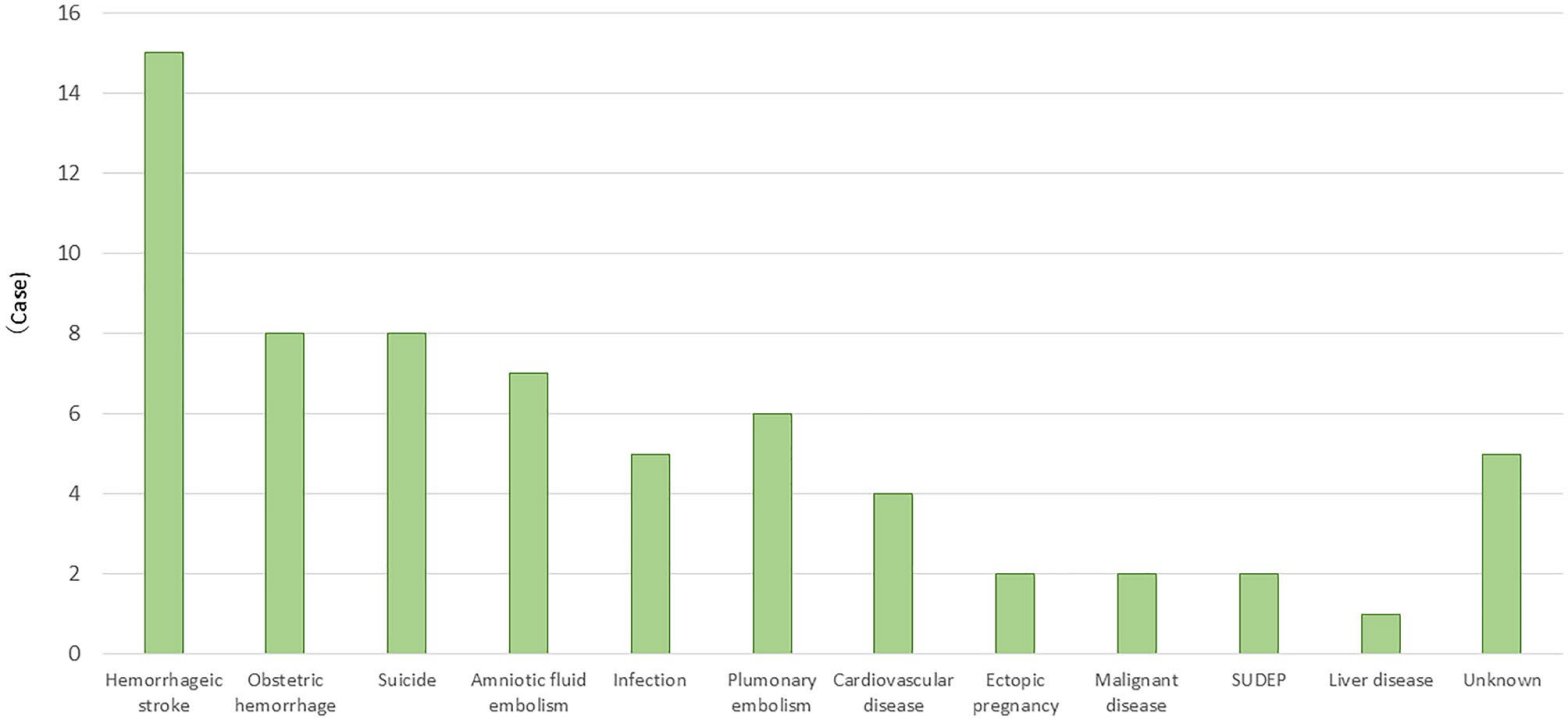
Causes of maternal mortality in those over 40 years of age.

**Figure 3:**
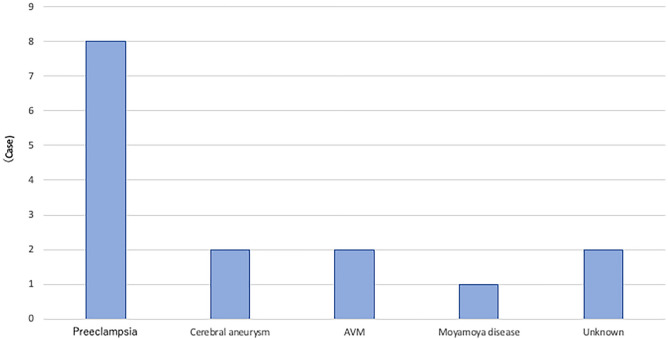
Causes of hemorrhagic stroke in maternal deaths among those over 40 years of age.

Table 2 and Fig. 2 shows the background data and the number of cases of maternal mortality among those aged ≥ 40 years. The most common cause of death was hemorrhagic stroke (15/65 [23%]), followed by obstetric hemorrhage (8/65 [12%]) and suicide (8/65 [12%]). The maternal mortality rates of hemorrhagic stroke, pulmonary thromboembolism, and suicide were significantly higher among those aged ≥ 40 years than among the other age groups (*P* < 0.01 for hemorrhagic stroke, pulmonary thromboembolism, and suicide). Figure 3 shows a breakdown of the causes of hemorrhagic stroke, the most common cause of death. The most common causes of hemorrhagic stroke were preeclampsia (8/15 [54%]), cerebral aneurysm (2/15 [13%]), cerebral arteriovenous malformation (2/15 [13%]), moyamoya disease (1/15 [7%]), and unknown (2/15 [13%]).

**Table 2:** Background of advanced maternal age (N = 65).

Age at delivery (years)	Case (%)
40	21 (32)
41	18 (28)
42	8 (12)
43	8 (12)
44	3 (5)
45	6 (9)
≥ 46	1 (2)
Parity	
0	23 (36)
1	13 (20)
2	15 (23)
3	6 (9)
≥ 4	6 (9)
Unknown	2 (3)
Conception	
Spontaneous	43 (67)
Assisted reproduction	7 (10)
Unknown	15 (23)
Multiple pregnancy	
Yes	1 (2)
No	64 (98)
Body mass index	
18–24	29 (45)
25–29	26 (40)
30–34	5 (7)
35–39	4 (6)
≥ 40	1 (2)
Any pre-existing medical or mental health problem	
Yes	16 (25)
No	49 (75)

## Discussion

This study is the first to report on the relationship between age and maternal mortality in Japan. Two important new findings were extracted from the present study. First, AMA in Japan was found to be associated with a significantly higher maternal mortality rate than that noted among pregnancies at a younger age in this study. Previous studies have also reported that an increase in maternal age is associated with an increase in various obstetric complications and maternal mortality^19–21^. The results of the present study are similar to those previously reported. However, Maternal mortality was notably lower among women younger than 20 years of age, a point of difference. Second, this is a clarification of the causes of maternal mortality among women over 40 years of age.

Although the rate of chronic diseases, such as malignant, cardiovascular, and renal diseases, as comorbidities is increased among AMA cases^22^, the rate of their involvement as a cause of death was low. The most common cause of death in the AMA group in this study was hemorrhagic stroke. The association of hemorrhagic stroke, rather than stroke, with maternal mortality is a problem unique to Asian populations^23,24^, and more than half of the deaths due to hemorrhagic stroke were associated with preeclampsia. In general, the overall incidence of preeclampsia is reported to be 3–4% but increases to 5–10% in those aged 40–49 years and to 35% in those aged > 50 years^25^. In addition, at ≥ 40 years, the relative risk of mortality was reported to be 1.68 (95% CI 1.23–1.39) for first-time mothers^26^. It is clear that the incidence of preeclampsia increases in AMA cases^27^. Older Japanese and Asian pregnant women are more likely to develop hemorrhagic stroke^28,29^, and adequate attention should be paid to the development of hemorrhagic stroke due to preeclampsia. Further, in Asian populations, uterine artery Doppler, angiogenesis factors, and aspirin therapy should be used to monitor and prevent the occurrence of hypertensive nephropathy in pregnant women > 40 years old.

Among assisted reproductive therapies, frozen-thawed embryo transfer and egg donation in particular have been reported to be risk factors for preeclampsia^30,31^. In the present study, the proportion of maternal mortality cases at age ≥ 40 years among those who had undergone assisted reproductive therapy was low. In addition, there were no cases of maternal mortality due to hemorrhagic stroke caused by preeclampsia among assisted reproductive therapy-induced pregnancy cases. The proportion of assisted reproductive technology-induced pregnancies in Japan is expected to increase in the future, as increasing age increases the dependence on assisted reproductive technology. Therefore, it is important to be aware of the possibility of developing preeclampsia after the use of assisted reproductive technology.

In addition to hemorrhagic stroke, pulmonary thromboembolism, infectious diseases, cardiovascular diseases, and suicide significantly increased maternal mortality in older pregnant women, suggesting that we should not focus solely on hemorrhagic stroke as a cause of maternal mortality.

This study showed that the maternal mortality rate increased with AMA in Japan. This is consistent with reports from other developed countries^3,32^. However, other developed countries have higher rates of maternal mortality among young women as well as among AMA cases, indicating a bimodal nature of this association, while the maternal mortality rate among women aged < 20 years in Japan was low. In the United Kingdom, the maternal mortality rate among pregnant women aged 20–29 years is the lowest, and the maternal mortality rates for pregnancies among those < 20 years of age and ≥ 30 years increased compared to those among pregnant women in their 20 s^3^. In addition, Japan provides generous medical insurance coverage to all citizens regardless of the financial status. During pregnancy, women are given a medical checkup slip almost free of charge and can visit a hospital every four weeks in the first trimester, every two weeks in the second trimester, and every week in the last trimester of pregnancy. Further, the groups enrolled in this study were almost exclusively Japanese, which provided a sample cohort that is less prone to biases other than age.

The limitations of this study include, first, that it was a mono-ethnic study and did not adjust the maternal mortality rates for several confounding factors, although the Japanese insurance system provides a background for the uniform provision of medical care. Second, although we had detailed data on deaths, we did not have detailed data on survivors. Third, we did not have information on hospital resources, infrastructure, and staff that contribute to maternal mortality and risk. It is not possible to determine whether high-risk women benefit from being in a particular healthcare setting. Fourth, because of the relatively low maternal mortality rates, the denominators for some of the groups were relatively small, making it impossible to make meaningful comparisons between the groups in terms of mortality rates.

In conclusion, maternal mortality increased proportionally with age in Japan. In particular, the mortality rate was 2.3 times higher for those aged ≥ 40 years than for those aged 35–39 years. In addition, hemorrhagic stroke was the most common cause of maternal mortality among those aged ≥ 40 years, and more than half of the hemorrhagic strokes were associated with preeclampsia. These facts should be considered by women who become pregnant at an advanced age and by healthcare providers involved in their perinatal care.

## Conclusion

Maternal mortality rates were significantly higher among older pregnant women than among younger pregnant women in Japan, with hemorrhagic stroke being the most common cause of maternal death among women > 40 years of age. More than half of the hemorrhagic strokes were associated with preeclampsia. These facts should be considered by women who become pregnant at an advanced age and by healthcare providers involved in their perinatal care.

## Data Availability

The data that support the findings of this study are available from the corresponding author, upon reasonable request.
